# The Volatile Chemical Compositions of the Essential Oil/SPME and Enzyme Inhibitory and Radical Scavenging Activities of Solvent Extracts and the Essential oils from *Coronilla orientalis* Miller and *C. varia* L. grows in Turkey

**DOI:** 10.22037/ijpr.2019.1100802

**Published:** 2019

**Authors:** Gülin Renda, Arzu Özel, Burak Barut, Büşra Korkmaz, Nurettin Yayli

**Affiliations:** a *Department of Pharmacognosy, Faculty of Pharmacy, Karadeniz Technical University, 61080, Trabzon, Turkey. *; b *Department of Biochemistry, Faculty of Pharmacy, Karadeniz Technical University, 61080, Trabzon, Turkey.*

**Keywords:** Essential oil, SPME-GC-MS, Acetylcholinesterase, Butyrylcholinesterase, Tyrosinase, *α*-Glucosidase, DPPH

## Abstract

The volatile organic compounds (VOCs) of two *Coronilla* species (*Coronilla orientalis* Miller and *Coronilla varia* L.) obtained by hydrodistillation (HD) and solid phase microextraction (SPME) techniques were identified by GC-FID/MS. The major compounds identified in the SPME extracts were limonene (43.4%) in *Coronilla orientalis (C. orientalis),* *(Z)-β-*ocimene and (*E*)-*β*-ocimene (34.3% and 32.4%) in *Coronilla varia (C. varia),* whereas, the essential oils of *C. orientalis* and *C. varia* were rich with *γ*-terpinene (22.4%) and phytol (30.7%), respectively. In addition, acetylcholinesterase (AChE), butyrylcholinesterase (BuChE), tyrosinase, *α*-glucosidase enzyme inhibitory, and radical scavenging activities (DPPH) of chloroform, ethyl acetate, methanol, and water extracts, and also essential oils obtained from *C. orientalis* and *C. varia* were investigated. The tyrosinase activity was studied at the doses of 25 µg/mL, 50 µg/mL and 100 µg/mL. Tyrosinase inhibition percentage was observed to increase by dose and methanol extracts of the both species were found to have the highest activity. Essential oils of the both species were found to have significant acetylcholinesterase and butyrylcholinesterase inhibition activities. *α*-Glucosidase enzyme inhibition of the ethyl acetate and water extracts of *C. orientalis* was determined as 80.11 ± 4.07% and 80.32 ± 3.47% at the 100 µg/mL concentration, respectively. Essential oils, chloroform, ethyl acetate, methanol, and water extracts were determined to have moderate DPPH radical scavenging activities.

## Introduction

Alzheimer disease is the most common form of dementia, leading to impaired cognitive function such as loss of memory, thinking ability, mutism, akinesia, and aphasia, therefore, it has become a major health issue in the developing countries (1). Although pathogenesis of Alzheimer disease is still not fully clear, the cholinergic hypothesis is the most accepted theory proposing that the disease is based on the degeneration of cholinergic neurons in the brain and associated with the loss of cholinergic neurotransmission in the cerebral cortex (2). Therefore, the treatment of this disease aims to enhance the choline level in the brain using cholinesterase inhibitors (3, 4). There are several cholinesterase inhibitors approved by Food and Drug Administration (FDA) in the United States, such as tacrine, donepezil, galantamine, and rivastigmine (5-8). Though, these compounds were reported to have side effects containing gastrointestinal problems, liver disorders, aggression, and depression (5-8).

Tyrosinase, a multifunctional copper-containing enzyme, plays an important role in melanin biosynthesis (9). An abnormal accumulation of melanin may trigger serious hyperpigmentation diseases of human skin, such as age spots, freckles, senile lentigines, melisma, and ephelides (10). In addition, Parkinson disease is one of the neurodegenarative diseases that affects the brain and is associated with overproduction of melanin. Researches on tyrosinase showed that it could contribute the neuromelanin formation in the human brain (11). Thus, the inhibitors of tyrosinase have potential applications in the cosmetic and medicinal area (12). 

Diabetes mellitus is a chronic disease, which occurs in the pancreas that does not generate enough insulin or of which cells do not reply to insulin that is produced (13). The management of glucose level in the blood is a crucial strategy for treatment of this disease. Thus, *α*-glucosidase inhibitors such as acarbose, miglitol, and voglibose are currently used against diabetes mellitus; however, some systematic adverse effects have been observed (14).

Reactive oxygen species (ROS) play an important role in oxidative injuries and cell damages. Thus, they lead to many diseases such as neurodegenerative, cardiovascular, cancer, diabetes, and gastrointestinal ulcergenesis. Plant-derived antioxidants, usually scavenging and reducing agents should be used to prevent or control the production of reactive oxygen species due to the presence of phenolic and volatile compounds (15).

Because of the effectiveness of natural products, researchers have been trying to search new natural inhibitors against these diseases. 

*Coronilla* L. (Fabaceae) genus is represented by 11 taxa of which, one is endemic in Turkey (16). Leaves and flowers of *Coronilla* species are used as cardiotonic and against cold, diabetes, kidney, and abdominal pains (17-21). Cardenolide glycosides, coumarins, proanthocyanidins and flavonoids were previously isolated from *Coronilla *species (22-27). *Coronilla varia* L. and *Coronilla orientalis *Miller are two of the *Coronilla* species naturally grown in Turkey (16).

Scopoletin, umbelliferone, *p*-coumaric acid, *o*-coumaric acid, leucoanthocyanins, catechin, ferulic acid, cyanidin, and delphinidin were isolated from the seeds of* C. varia* previously (28). Additionally, some flavonoids such as kaempferol, astragalin, trifolin, saponaretin, and homoorientin were isolated from the aerial parts of *C. varia.* Robiinin was found as the most potent substance in terms of hypoazotemic activity (29). Cardenolides, coumarins, and 3-nitropropionates of glucose were isolated from *C. varia*, and tested for their insect antifeedant activity. The highest anti-feeding activity was reported to be observed with 3-nitropropionic acid (30). Similarly, alcoholic extract of the seeds of *C. varia* showed inhibitory activity against KB cells and further studies led to get hyrcanoside, daphnoretin, scopoletin, and umbelliferone (31). Hyrcanoside and deglucohyrcanoside which were isolated from the seeds of *C. varia*, were reported to show remarkable cardiotonic activity (Na+, K+- ATPase inhibitory activity) (32). Antibacterial and antitumor activities were also screened for water, ethanol, and methanol extracts of *C. varia.* Ethanolic and methanolic extracts *C. varia* were mentioned to have strong antibacterial activity against gram-negative bacteria (33).

Similar chemical compounds were isolated from the seeds of *C. orientalis* when compared with *C. varia*. Both were reported to contain coumarins and cardiac glycosides; hyrcanoside, hyrcanogenin, and the Δ4-isomer of 5-anhydrostrophanthidin (34). Also, umbelliferone, scopoletin, and daphnoretin were isolated from the seeds of *C. orientalis* (35). 

Coumarins, phenolic and volatile compounds which were dominant in *Coronilla* species, as mentioned above, are the phytochemicals with wide range of biological activities including AChE inhibition (15, 36). The literature survey showed that chemical composition of essential oils and enzyme inhibition and radical scavenging activities of* C. orientalis *and* C. varia* grown in Turkey has not been investigated previously. Hence, the aim of this study was to identify the volatile chemical composition and evaluate the acetylcholinesterase, butyrylcholinesterase, tyrosinase, *α*-glucosidase inhibition behaviors, and 2,2-diphenyl-1-picrylhydrazyl (DPPH) radical scavenging activities of essential oils and chloroform, ethyl acetate, methanol, and water extracts were obtained from two *Coronilla *species; *C. varia* and *C. orientalis.*

## Experimental

*Plant materials*

*Coronilla orientalis* Miller and *Coronilla varia* L. species were collected from Çeşmeler village, Maçka, Trabzon, Turkey, on May 2015. Taxonomic identification of the plant materials was made by Dr Gülin Renda according to the Flora of Turkey and the East Aegean Islands (17). The voucher specimens of the *C. varia* and *C. orientalis* were deposited at the Hacettepe University Faculty of Pharmacy Herbarium (Voucher No: HUEF 15008 and 15009 respectively).

*Chemicals and reagents*

Acetylcholinesterase enzyme (AChE), acetylthiocholine iodide, butyrylcholinesterase (BuChE), butyrylthiocholine iodide, 5,5′-dithio-bis(2-nitrobenzoic acid) (DTNB), galantamine, tyrosinase from mushroom, L-DOPA, kojic acid, *α*-glucosidase from *Saccharomyces cerevisiae*, acarbose, *p*-nitrophenyl-*α*-glucopyranoside, Folin–Ciocalteu reagent and 2,2-diphenyl-1-picrylhydrazyl (DPPH) were purchased from Sigma-Aldrich. Ultra-HPLC grade water was used. All other solvents were purchased from Sigma-Aldrich.

*Isolation of essential oils*

The whole parts of the air dried two *Coronilla* species (100 g, per each species) were hydrodistilled in a modified Clevenger-type apparatus separately using cooling bath (-15 ºC) system (3 h). The essential oils (HD) (1.210 g and 0.083 g, respectively) were taken and dissolved in HPLC grade *n*-hexane (0.5 mL) and kept at 4 ºC in a sealed brown vial until analysis. 

*SPME analysis*

Each airdried plant material (1 g) was grounded and placed in a vial for SPME analysis. Vials were in 10 mL volume and sealed with a silicone-rubber septum cap. A polydimethylsiloxane/divinylbenzene fiber was used for the extraction of the volatile components. Extractions were achieved with magnetic stirring. The fiber coating was placed to the head space and before the analysis, the fibers were conditioned for 5 min at 250 °C in GC injector. Temperature, incubation and extraction times were set according to the experiment. SPME was done at 50 °C with incubation time of 5 min, and extraction time of 10 min. Each sample was analyzed and reported. 

*Gas chromatography-mass spectrometry (GC/MS)*

The gas chromatography-flame ionization detector (GC-FID) analysis was carried out on a Shimadzu QP2010 plus gas chromatography coupled to a Shimadzu QP2010 Ultra mass selective detector. The fiber containing the extracted aroma compounds were injected into the GC-MS injector (split mode). Separation took place with a Restek Rxi-5MS capillary column, 30 m length, 0.25 mm i.d., and 0.25 μm phase thickness. The oven program was as follows: initial temperature was 60 °C for 2 min, which was increased to 240 °C at 3 min, final temperature 250 °C was held for 4 min. Injector temperature was 280 °C; split ratio, 1:40. The carrier gas was helium (99.999%), at a constant flow rate 1 mL/min; sample size, 0.5 µL. The detection was carried out in electronic impact mode (EI); ionization voltage was fixed to 70 eV, Scan mode (40-450 m/z) was used for mass acquisition (37).

*Compound identification*

The volatile compounds were identified by comparison of their retention indices and mass spectra with those of the mass spectra of the two libraries (FFNSC1.2 and W9N11).

*Extraction*

The whole grounded airdried plant of *C. orientalis* and *C. varia* (15 g and 20 g, each species) were extracted with chloroform, ethyl acetate, methanol, and water solvents using a rotary evaporator for 2 h (300 mL × 2 times) at 40 °C, for each sample, respectively. The extracts were filtered and the solvents were removed under vacuum by using a rotary evaporation to give chloroform (0.317 g, 0.292 g), ethyl acetate (0.335 g, 0.225 g) methanol (1.067 g, 0.792 g), and water (0.932 g, 1.047 g) crude extracts, respectively, which were kept in a refrigerator at +4 °C until used. Crude chloroform, ethyl acetate, methanol, water extracts (100 mg each extract), and essential oils (0.170 g, 0.083 g, respectively) (HD) of *C. orientalis* and *C. varia* were investigated for the enzyme inhibitory and radical scavenging activities.

*Enzyme inhibitions*

*Acetylcholinesterase/Butyrylcholinesterase (AChE/BuChE) inhibition*

The acetylcholinesterase/butyrylcholinesterase (AChE/BuChE) inhibition was examined using the method described by Ellman (38) and Ingkaninan (1). Galantamine was used as the reference drug. Fifty microliters of 50 mM Tris-HCl buffer (pH 8.00), 125 µL of 3 mM DTNB (in buffer), 25 µL of 0.2 U/mL AChE/BuChE and 25 µL of extracts at the concentrations of 25 µg/mL, 50 µg/mL and 100 µg/mL were added in a 96-well microplate. The mixtures were incubated for 15 min at 25 °C. After incubation, 25 µL of 15 mM acetylthiocholine iodide/butyrylthiocholine iodide was added in microplate. After incubation for 5 min at room temperature, the absorbance was measured using a 96-well microplate reader at 412 nm. Calculation of the inhibition of AChE/BuChE was done by using the Equation 1. A_control_ is the activity of enzyme without extract (solvent in buffer pH 8) and A_sample_ is the activity of enzyme with extract at different concentrations. 

Equation 1:           $\mathrm{Inhibition}(\%)=\left[\frac{\left(A_{\mathrm{control}}-A_{\mathrm{sample}}\right)}{A_{\mathrm{control}}}\right]\times 100$

*Tyrosinase inhibition*

The tyrosinase inhibition was examined using the method described by Masuda (39). Kojic acid was used as the reference drug. Twenty microliters of the extracts at the concentrations of 25 µg/mL, 50 µg/mL and 100 µg/mL, 20 µL of 250 U/mL tyrosinase and 100 µL of 100 mM pH 6.8 phosphate buffer solutions were added in a 96-well microplate. The reaction was initiated with addition of 20 µL of 3 mM *L*-DOPA and the absorbance was measured at 475 nm using a 96-well microplate reader. The tyrosinase inhibition percentage was calculated using the Equation 1.

*α-Glucosidase inhibition*

The* α*-glucosidase inhibition was examined using the method described by da Silva Pinto (40). Acarbose was used as the reference drug. Fifty microliters of extracts at different concentrations (25 µg/mL, 50 µg/mL and 100 µg/mL) with 100 µL of 0.5 U/mL *α*-glucosidase enzymes was added in a 96-well microplate. The mixtures were incubated for 15 min at 25 °C. After incubation, 50 µL of 5 mM *p*-nitrophenyl-*α*-glucopyranoside was added and incubated at 25 °C for 10 min. The absorbance was measured at 405 nm using a 96-well microplate reader. The *α*-glucosidase inhibition percentage was calculated using the Equation 1. 

*Antioxidant activity*

*2,2-Diphenyl-1-picrylhydrazyl (DPPH) Radical Scavenging Assay*

The 2,2-diphenyl-1-picrylhydrazyl (DPPH) radical scavenging activities were examined using the method described by Blois compared to gallic acid and ascorbic acid as the reference compounds (41). Total volume of assay mixture which was 1 mL, contained methanolic DPPH solution (0.4 mM) and different concentrations (25 µg/mL, 50 µg/mL and 100 µg/mL) of extracts. The mixtures were incubated for 30 min at room temperature in the dark. After incubation, the absorbance of the sample (A_sample_) was measured at 517 nm. Assay mixture without samples was used as a control (A_control_). The inhibition percentage was calculated using the Equation 2. 

Equation 2:            $\mathrm{Scavenging effects}(\%)=\left[\frac{\left(A_{\mathrm{control}}-A_{\mathrm{sample}}\right)}{A_{\mathrm{control}}}\right]\times 100$

*Statistical Analysis*

The experiments were carried out in triplicate and results were expressed as the mean ± standard deviation (SD). The statistical analysis was performed with SPSS 15.0 for Windows and Microsoft Excel for Windows 10. The differences among the extracts were evaluated by One-way analysis of variance (ANOVA) followed by Duncan’s multiple range tests. *P* < 0.05 was considered statistically significant.

## Results

*Chemical composition*

The hydrodistillation of the whole part of air dried* C. orientalis *and* C. varia* gave yellowish oils with a yield of 0.170% (w/w) and 0.083% (w/w), respectively. Composition of the volatile organic compounds for the HD and SPME of *C. orientalis *and* C. varia* were characterized with GC-FID/MS and the results were given in Table 1 and their chemical class distributions are given in Table 2. A total of 18 and 8 compounds comprising 99.8% and 99.9% of the HD and SPME of *C. orientalis* and 16 and 14 compounds comprising 99.9% and 99.8% of the HD and SPME of *C. varia* were identified, respectively (Tables 1 and 2).

Results of SPME and HD analysis within the species were highly different. Hexanal (9.1% and 1.1%, respectively) and linalool (15.6% and 8.8%, respectively) were the only compounds which were present both in the HD and SPME of *C. orientalis. *Results showed that, components of *C. varia *which were detected with HD and SPME analysis, were totally different.

γ-terpinene (22.4%) and linalool (15.6%) were the main components which were detected in *C. orientalis* essential oil with HD. Whereas limonene (43.4%) was the main component identified within the same species by SPME method. Also SPME analysis has shown 8.8% of linalool in *C. orientalis* (Table 1).

GC-MS analysis of HD essential oil of *C. varia* revealed the presence of phytol (30.7%) and tricosane (24.9%) as the major compounds. Whereas (*Z*)-*β*-Ocimene (34.3%) and (*E*)-*β*-Ocimene (32.4%) in *C. varia* were the main compounds identified by SPME method. 

Monoterepene hydrocarbons were the major constituents in the essential oil of *C. orientalis *detected both with HD (23.5%) and SPME (43.4%) analysis of *C. orientalis.* Also *C. varia* reavelad the presence of monoterepene hydrocarbons as the main components just detected with SPME analysis within the ratio of 72.3%. Oxygenated sesquiterpenes were detected in both species only with HD analysis. While monoterpene hydrocarbons and oxygenated monoterpenes of *C. varia* were only found with SPME analysis (Table 2).

*Enzyme inhibitory activity*

The enzyme inhibition and radical scavenging activities for the essential oils (EO) and chloroform, ethyl acetate, methanol and water extracts of* C. orientalis *(CO) and* C. varia *(CV) were tested *in-vitro* (Tables 3-7). In this work, different concentrations (25 μg/mL, 50 μg/mL, and 100 μg/mL) of the chloroform (CO/CV-CEx), ethyl acetate (CO/CV-EAEx), methanol (CO/CV-MEx) and water (CO/CV-WEx) extracts of *C. orientalis *and* C. varia* were used to determine acetylcholinesterase, butyrylcholinesterase, tyrosinase, and *α*-glucosidase inhibition activities, respectively. 

The AChE/BChE inhibitory potential of extracts and essential oils were presented in Tables 3 and 4, as inhibition (%) values for each concentration. The results indicated that the extracts and essential oils inhibited AChE/BuChE enzymes in a dose dependent manner. COEO had the highest AChE inhibiton among the other extracts with 59.11 ± 3.29%, 65.64 ± 2.83% and 72.23 ± 2.66%, at the doses of 25 μg/mL, 50 μg/mL and 100 μg/mL, respectively, and it also gave the highest BuChE inhibition with 61.44 ± 3.51%, 69.48 ± 2.81% and 76.44 ± 4.03%, at the doses of 25 μg/mL, 50 μg/mL and 100 μg/mL, respectively. Essential oils of *C. orientalis *and* C. varia* at 100 μg/mL concentration inhibited highly AChE and BuChE enzymes activities with 67.43 ± 1.81% and 69.77 ± 3.27%, whereas, CVWEx did not inhibit AChE enzyme at concentrations of 25 μg/mL or 50 μg/mL.

The results of the tyrosinase inhibition of the extracts and the essential oils were expressed as inhibition (%) values and presented in Table 5. The results revealed that the extracts and the essential oils inhibited tyrosinase enzyme in a dose dependent manner. As shown in Table 5, high levels of tyrosinase inhibition observed in CVMEx (45.89 ± 1.46% and 52.91 ± 2.82%) with 50 μg/mL and 100 μg/mL, respectively. However, COMEx had the most potent with the range of 41.68 ± 1.88% at 25 μg/mL. On the other hand, CVCEx showed the lowest inhibitory effect in the range of 16.47 ± 1.53% - 34.31 ± 1.94%.

*α*-glucosidase inhibitory effects of the extracts and the essential oils were expressed as inhibition (%) values and summarized in Table 6. COWEx had significantly (*P* < 0.05) high inhibitory effect among the other extracts with 80.32 ± 3.47% at 100 μg/mL. CO/CV had significantly (*P* < 0.05) high* α*-glucosidase inhibition at all of the tested concentrations. On the other hand, CVWEx were inactive against *α*-glucosidase enzyme at 25 μg/mL and 50 μg/mL. When the concentration of COEAEx ranged from 25 μg/mL to 100 μg/mL, the percentage of *α*-glucosidase inhibition ranged from 22.67 ± 1.68% to 80.11 ± 4.07%. 

*Antioxidant capacity*

The radical scavenging activities of extracts and essential oils were determined using 2,2-diphenyl-1-picrylhydrazyl (DPPH) radical scavenging assay. These results were presented in Table 7. All of tested extracts and the essential oils indicated moderate scavenging activities, compared to gallic acid as the reference compound for this assay. COMEx showed scavenging activities in the range of 12.59 ± 0.11% - 41.91 ± 0.97% at all of the tested concentrations, whereas CVMEx showed scavenging activities in the range of 9.89 ± 0.03% - 39.40 ± 1.63% at all of the tested concentrations, respectively. In addition, CO/CV scavenged the DPPH radical in a dose dependent manner with 14.77 ± 0.22%, 26.77 ± 0.09%, and 38.50 ± 1.22%, at the concentrations of 25 μg/mL, 50 μg/mL, and 100 μg/mL respectively.

**Table 1:** Volatile organic compounds of the *C. orientalis *and *C. varia *from Turkey

**Compunds**	**Lit.RI**	**RI** **a**	**RT**	**CO-HD (%)** **b**	**CO-SP (%)** **b**	**CV-HD (%)** **b**	**CV-SP (%)** **b**
Methylbenzene	779	781	7.94	-	-	19.0	-
Hexanal	802	802	8.57	9.1	1.1	-	-
(2*E*)-Hexenal	846	848	10.14	4.5	-	-	-
(3*Z*)-Hexenol	850	851	10.24	1.3	-	-	0.3
(2*E*)-Hexenol	854	861	10.56	0.6	-	-	-
Octen-3-ol	974	976	14.69	5.2	-	-	-
Benzonitrile	990	988	15.18	-	-	1.8	-
Myrcene	991	992	15.316	-	-	-	2.7
Octanal	998	1003	15.791	-	-	-	0.7
(3*E*)-Hexenyl acetate	1001	1006	16.005	-	1.1	-	-
*p*-Cymene	1025	1025	16.720	1.1	-	-	-
Limonene	1029	1032	17.039	-	43.4	-	0.6
*β *-Phellandrene	1030	1033	17.068	-	-	-	0.3
*(Z)-β-*Ocimene	1037	1037	17.241	-	-	-	34.3
(*E*)-*β*-Ocimene	1044	1047	17.705	-	-	-	32.4
*γ*-Terpinene	1060	1058	18.161	22.4	-	-	-
Linalool	1097	1097	19.835	15.6	8.8	-	11.8
Nonanal	1101	1103	20.120	-	3.2	-	1.8
*Allo*-Ocimene	1128	1128	21.203	-	-	-	0.6
Terpineol	1134	1131	21.330	-	-	-	3.4
*Allo*-n*eo-*Ocimene	1140	1141	21.761	-	-	-	1.4
Terpinen-4-ol	1174	1178	23.374	1.7	-	-	-
Naphthalene	1181	1180	23.906	-	19.4	-	6.5
*(E)*-Caryophyllene	1408	1411	32.946	2.1	-	-	-
*α-(E)-*Bergamotene	1432	1429	33.978	-	13.1	-	-
*(E)*-Ethyl cinnamate	1465	1468	35.106	-	9.8	-	3.0
Pentadecane	1500	1500	35.571	-	-	2.7	-
Isopropyl dodecanoate	1615	1619	40.640	-	-	0.8	-
*α-*Muurolol	1640	1636	41.581	5.9	-	-	-
*δ-*Cadinol	1644	1648	41.652	4.5	-	-	-
*α-*Cadinol	1652	1650	42.085	2.8	-	-	-
Hexenyl salicylate	1669	1668	42.328	-	-	1.9	-
*α-*Bisabolol	1685	1685	42.936	-	-	2.0	-
Pentadecanal	1713	1708	44.340	-	-	0.1	-
Myristic acid	1748	1751	45.163	2.0	-	0.4	-
Benzyl benzoate	1759	1759	45.755	-	-	1.0	-
6,10,14-Trimethyl-2- pentadecanone	1847	1847	47.968	7.0	-	-	-
Hexahydrofarnesyl acetone	1846	1848	47.973	-	-	7.4	-
Palmitic acid	1959	1962	51.544	3.4	-	4.8	-
Phytol	2112	2113	56.287	-	-	30.7	-
Linoleic acid	2132	2131	56.790	2.8	-	0.5	-
Linolenic acid, methyl ester	2139	2143	56.837	-	-	1.2	-
Pregnane	2175	2178	58.375	-	-	0.7	-
Tricosane	2300	2296	61.069	7.8	-	24.9	-
Total		-	-	99.8	99,9	99.9	99.8

aRetention Index calculated from retention times relative to that of n-alkane series.

bPercentages obtained by FID peak-area normalization; CO-HD: hydrodistillation *of C. orientalis*; CO-SP: SPME of *C. orientalis*; CV-HD: hydrodistillation *of C. varia*; CV-SP: SPME of *C. varia.*

**Table 2:** The chemical class distribution of the essential oils and SPME components of *Coronilla *species

***C. orientalis *** **HD**			***C. orientalis *** **SPME**		***C. varia *** **HD**		***C. varia *** **SPME**	
	**Area (%)**	**NC** **a**	**Area (%)**	**NC** **a**	**Area (%)**	**NC** **a**	**Area (%)**	**NC** **a**
Monoterpene hydrocarbons	23.5	2	43.4	1	-	-	72.3	7
Oxygenated monoterpenes	17.3	2	8.8	1	-	-	15.2	2
Sesquiterpene hydrocarbons	2.1	1	13.1	1	-	-	-	-
Oxygenated sesquiterpenes	13.2	3	-	-	2.0	1	-	-
Aldehydes	13.6	2	4.3	2	0.1	1	2.5	2
Esters	-	-	10.9	2	4.9	4	3.0	1
Alcohol	7.1	3	-	-	30.7	1	0.3	1
Others	23.0	5	19.4	1	62.2	9	6.5	1
Total	99.8	18	99.9	8	99.9	16	99.8	14

aNC: Number of compounds.

**Table 3:** The acetylcholinesterase inhibitory effects of solvent extracts and essential oils obtained from *C. orientalis *and *C. varia*

**Acetylcholinesterase inhibitory activity (Inhibition% ± SD** **a** **)**						
**µg/mL**	**25**		**50**		**100**	
	**CO**	**CV**	**CO**	**CV**	**CO**	**CV**
Essential oil	59.11 ± 3.29	40.33 ± 1.51	65.64 ± 2.83*	47.81 ± 2.23	72.23 ± 2.66*	67.43 ± 1.81
Chloroform	19.19 ± 0.37	6.31 ± 0.20	27.17 ± 2.15	12.47 ± 1.01	37.91 ± 2.68	26.72 ± 1.36
Ethyl acetate	16.20 ± 0.73	5.27 ± 0.34	24.74 ± 1.89	12.79 ± 0.86	33.88 ± 2.94	18.13 ± 0.81
Methanol	10.47 ± 0.73	7.74 ± 0.42	24.59 ± 1.52	10.08 ± 1.86	31.36 ± 2.15	14.08 ± 1.02
Water	5.91 ± 0.41	ND	8.07 ± 0.56	ND	18.08 ± 1.36	6.38 ± 0.82
Galantamine	82.89 ± 0.22		83.81± 0.53		85.41 ± 0.51	

CO: *C. orientalis*; CV: *C. varia*; aSD: Standard deviation (n = 3). ND: No activity. *: (*P *< 0.05).

**Table 4:** The butyrylcholinesterase inhibitory effects of solvent extracts and essential oils obtained from *C. orientalis *and *C. varia*

**Butyrylcholinesterase inhibitory activity (Inhibition% ± SD** **a** **)**						
**µg/mL**	**25**		**50**		**100**	
	**CO**	**CV**	**CO**	**CV**	**CO**	**CV**
Essential oil	61.44 ± 3.51	49.88 ± 1.95	69.48 ± 2.81	60.41 ± 1.46	76.44 ± 4.03	69.77 ± 3.27
Chloroform	30.17 ± 1.48	15.64 ± 0.73	37.99 ± 1.57	25.50 ± 0.92	53.49 ± 2.52	32.89 ± 1.77
Ethyl acetate	20.30 ± 0.84	3.29 ± 0.31	26.04 ± 1.30	13.30 ± 0.36	32.45 ± 1.15	23.02 ± 0.73
Methanol	9.87 ± 0.22	3.70 ± 0.19	18.65 ± 0.84	9.88 ± 0.86	27.31 ± 1.73	17.40 ± 1.88
Water	7.68 ± 0.36	ND	12.75 ± 0.65	6.44 ± 0.27	18.67 ± 1.57	12.20 ± 0.94
Galantamine	75.56 ± 0.44		78.03 ± 0.21		80.32 ± 0.23	

CO: *C. orientalis*; CV: *C. varia*; aSD: Standard deviation (n = 3). ND: No activity.

**Table 5:** The tyrosinase inhibitory effects of solvent extracts and essential oils obtained from *C. orientalis *and *C. varia*

**Tyrosinase inhibitory activity (Inhibition% ± SD** **a** **)**						
**µg/mL**	**25**		**50**		**100**	
	**CO**	**CV**	**CO**	**CV**	**CO**	**CV**
Essential oil	20.84 ± 0.91	18.48 ± 0.45	38.68 ± 2.04	28.06 ± 1.91	45.49 ± 1.36	39.87 ± 2.44
Chloroform	19.68 ± 0.36	16.47 ± 1.53	26.47 ± 1.42	25.47 ± 3.61	38.69 ± 2.68	34.31 ± 1.94
Ethyl acetate	33.27 ± 1.03	30.67 ± 2.14	36.27 ± 2.75	35.04 ± 1.63	48.09 ± 3.66	42.08 ± 2.41
Methanol	41.68 ± 1.88	38.47 ± 0.53	45.66 ± 2.28	45.89 ± 1.46	52.90 ± 3.25	52.91 ± 2.82
Water	26.27 ± 1.79	20.27 ± 0.90	40.70 ± 1.69	28.67 ± 1.62	48.11 ± 2.93	35.04 ± 1.72
Kojic Acid	80.09 ± 0.19		84.20 ± 0.22		85.63 ± 0.09	

CO: *C. orientalis*; CV: *C. varia*; aSD: Standard deviation (n = 3).

**Table 6:** The *α*-glucosidase inhibitory effects of solvent extracts and essential oils obtained from *C. orientalis *and *C. varia*

***α*** **-glucosidase inhibitory activity (Inhibition% ± SD** **a** **)**						
**µg/mL**	**25**		**50**		**100**	
	**CO**	**CV**	**CO**	**CV**	**CO**	**CV**
Essential oil	25.04 ± 1.22*	21.35 ± 0.55	36.59 ± 2.47*	31.47 ± 1.82	70.25 ± 3.86*	59.63 ± 2.11
Chloroform	26.00 ± 0.29	18.34 ± 0.73	36.02 ± 1.36	24.44 ± 0.66	57.97 ± 1.93	44.46 ± 0.91
Ethyl acetate	22.67 ± 1.68	14.89 ± 1.74	40.22 ± 2.59	21.52 ± 0.79	80.11 ± 4.07*	37.77 ± 2.19
Methanol	17.38 ± 0.37	8.30 ± 0.13	31.93 ± 1.72	15.35 ± 0.57	62.86 ± 2.74	30.91 ± 1.31
Water	22.11 ± 1.04	ND	42.98 ± 2.82	ND	80.32 ± 3.47*	12.04 ± 0.35
Acarbose	62.09 ± 0.20		65.20 ± 0.02		73.63 ± 0.04	

CO: *C. orientalis*; CV: *C. varia*; aSD: Standard deviation (n = 3). ND: No activity. *: (*P *< 0.05).

**Table 7:** The 2,2-diphenyl-1-picrylhydrazyl (DPPH) radical scavenging activities of solvent extracts and essential oils obtained from *C. orientalis *and *C. varia*

**DPPH radical scavenging activities (Inhibition% ± SD** **a** **)**						
**µg/mL**	**25**		**50**		**100**	
	**CO**	**CV**	**CO**	**CV**	**CO**	**CV**
Essential oil	14.77 ± 0.22	13.64 ± 0.30	26.77 ± 0.09	26.59 ± 0.66	38.50 ± 1.22	36.27 ± 0.83
Chloroform	16.09 ± 0.08	6.43 ± 0.14	22.00 ± 0.35	8.94 ± 0.20	37.34 ± 1.31	14.90 ± 0.27
Ethyl acetate	15.81 ± 0.12	11.83 ± 0.52	25.03 ± 0.38	18.97 ± 0.33	37.48 ± 0.77	27.73 ± 0.59
Methanol	12.59 ± 0.11	9.89 ± 0.03	16.85 ± 0.05	22.73 ± 0.80	41.91 ± 0.97*	39.40 ± 1.63
Water	8.14 ± 0.08	8.64 ± 0.02	10.55 ± 0.84	14.90 ± 0.51	20.45 ± 0.27	27.31 ± 0.42
Gallic Acid	95.40 ± 0.01		95.46 ± 0.04		96.24 ± 0.06	

CO: *C. orientalis*; CV: *C. varia*; aSD: Standard deviation (n = 3). ND: No activity. *: (*P *< 0.05).

## Discussion

The plants are valuable sources of new biologically active molecules due to the large numbers of secondary metabolites. New natural compounds or plant extracts possessing high enzyme inhibitory activity can be evaluated as a source of new agents for contemporary diseases. From this point of view, we decided to investigate acetylcholinesterase,butyrylcholinesterase, tyrosinase, *α*-glucosidase inhibition behaviors and 2,2-diphenyl-1-picrylhydrazyl (DPPH) radical scavenging activities of two *Coronilla* species which are utilized as forage crops besides their usage in Turkish folk medicine. To the best of our knowledge, this is the first evaluation of the enzyme inhibitory activities and GC-MS analysis of *C. orientalis* and *C. varia*. The extracts were prepared with four different solvents and the essential oils were obtained by hydrodistillation for the activity studies.

Methanol extracts of both species exhibited the highest DPPH radical scavenging activities (Table 7). As mentioned in the literature, phenolic compounds may be responsible for the biological activities of the species. Currently, the ethyl acetate and water extracts of *C. orientalis* was found to have the highest *α*-glucosidase inhibitory effect (Table 6). The tests of α-glucosidase inhibitory activity *in-vitro* and *in-vivo* may not always correlate with each other. So, it is necessary to confirm the activity within the *in-vivo* test systems. 

The enzyme inhibitory results showed that among the extracts, the essential oil of *C. orientalis* exhibited the most potent AChE inhibitory activity. The inhibition% values of the essential oil of *C. varia* were also closer to *C. orientalis* (Tables 3 and 4). There are several different studies that focused on the AChE and BuChE inhibitory activities of the essential oils in the literature (42). In a similar way, *C. varia* essential oil which contained monoterpene hydrocarbones as the main group, remarkably inhibited AChE and BuChE (Tables 1 and 2). It has been reported that the inhibitory activity of essential oils was mainly due to the presence of terpenoids especially monoterpenoids (42, 43).

Because of the remarkable activity of essential oils, the VOCs compositions of the *C. orientalis* and *C. varia *were investigated (Tables 1 and 2). Since the essential oils are complex mixtures of volatile compounds, there are varied extraction procedures for the characterization of essential oils (44-46). A solid phase microextraction (SPME) method was used besides hydrodistillation (HD) method for the analysis of the volatile compounds. 

The major constituent for the SPME of *C. orientalis* was found as limonene (43.4%) (Table 1) which was the only monoterpene hydrocarbon found in it (Table 2). Earlier search on the analysis of the volatile organic compounds of *Coronilla valentina* Pall. ex Bieb. growing in Mediterranean had (*E*)-*β*-ocimene as the major constituents (47). Also, it has been reported that the class of volatile organic compounds of *C. valentina* was rich in monoterpene hydrocarbons (48). This data set was also confirmed by our experimental results.

The chemical composition of essential oil of *C. varia* which was collected from North of Iran, was reported to contain caryophyllene oxide (60.19%), *α*-cadinol (4.13%), and homoadantaneca robexylic acid (3.31%) as major compounds (49). Caryophyllene oxide and homoadantaneca robexylic acid are not detected with our experiments. And in our results, *α*-cadinol was found in small amounts (2.8%) only in *C. orientalis.*

Although several components were detected in the HD oil of the both species, few compounds were detected by SPME according to HD. This could be explained the mechanisms of the methods. HD extracts the essential oil of the plant, whereas SPME extracts the volatile compounds present in the headspace of the leaves. The differences between HD and SPME gives lead us to achieve results on a wider scale because the SPME method exploits mild experimental conditions, whereas HD occurs at approximately the boiling point of water (50).

The relative composition obtained by SPME analysis shows a higher diversity of monoterpenes on the essential oil profiles of both species comparing with HD. These results suggest most of the highly volatile components absent in HD and SPME of the species were lost due to the extended times with high temperature during the extraction process (51). 

In conclusion, by determining the enzyme inhibitory and radical scavenging activities of two *Coronilla* species and determining the chemical composition of the essential oil and SPME analysis, we showed that *Coronilla* species could be evaluated as a source of natural agents. Especially, the essential oil of *C. orientalis* found to have a promising inhibitory potential against the tested neurodegeneration related enzymes. Thus, the results indicate the requirement of further investigations could be done for the essential oil of *C. orientalis* due to the medicinal purposes. Altogether, the results obtained from this study may also help to design further studies on the identification of individual bioactive constituents. 
